# Employing Titanium Dioxide Nanoparticles as Biostimulant against Salinity: Improving Antioxidative Defense and Reactive Oxygen Species Balancing in Eggplant Seedlings

**DOI:** 10.3390/antiox13101209

**Published:** 2024-10-08

**Authors:** Muhammad Fasih Khalid, Muhammad Zaid Jawaid, Muddasir Nawaz, Rana Abdul Shakoor, Talaat Ahmed

**Affiliations:** 1 Environmental Science Center, Qatar University, Doha 2713, Qatar; mfasih.khalid@qu.edu.qa (M.F.K.); mk1208853@student.qu.edu.qa (M.Z.J.); 2 Center for Advanced Materials, Qatar University, Doha 2713, Qatar; m.nawaz@qu.edu.qa (M.N.); shakoor@qu.edu.qa (R.A.S.); 3 Department of Mechanical and Industrial Engineering, Qatar University, Doha 2713, Qatar

**Keywords:** antioxidative enzymes, reactive oxygen species, nanotechnology, sodium toxicity, vegetables

## Abstract

Salinity is a major abiotic stress that affects the agricultural sector and poses a significant threat to sustainable crop production. Nanoparticles (NPs) act as biostimulants and significantly mitigate abiotic stress. In this context, this experiment was designed to assess the effects of foliar application of titanium dioxide (TiO_2_) nanoparticles at 200 and 400 ppm on the growth of eggplant (*Solanum melongena*) seedlings under moderate (75 mM) and high (150 mM) salinity stress. The TiO_2_-NPs employed were characterized by X-ray diffraction (XRD), Fourier transform infrared spectroscopy (FTIR), thermal gravimetric analysis (TGA), and scanning electron microscopy (SEM) analysis. The seedlings were assessed physiologically, growth-wise, and biochemically. The seedlings were significantly affected by their physiological attributes (Fv′/Fm′, Fv/Fm, NPQ), growth (root length, shoot length, number of leaves, fresh biomass, dry biomass, leaf greenness), antioxidative enzymes (SOD, POD, CAT, APx, GR), stress indicators (H_2_O_2_, MDA), and toxic ion (Na^+^) concentrations. The maximum decrease in physiological and growth attributes in eggplant seedling leaves was observed with no TiO_2_-NP application at 150 mM NaCl. Applying TiO_2_-NPs at 200 ppm showed significantly less decrease in Fv’/Fm’, root length, shoot length, number of leaves, fresh biomass, dry biomass, and leaf greenness. In contrast, there were larger increases in SOD, POD, CAT, APx, GR, and TSP. This led to less accumulation of H_2_O_2_, MDA, and Na^+^. No significant difference was observed in higher concentrations of TiO_2_-NPs compared to the control. Therefore, TiO_2_-NPs at 200 ppm might be used to grow eggplant seedlings at moderate and high salinity.

## 1. Introduction

Salinity is a significant abiotic stress that constantly threatens crop production and global food security. Currently, about 23% of farmlands and 25–30% of irrigated areas are affected by salt stress [1]. Due to urbanization and increased food demand, the move to drier farmland reduces land and water resources. Salinity stress affects plants by decreasing the soil’s osmotic ability, causing nutritional imbalances, and increasing ionic toxicity [2]. The high salinity in the soil is causing a significant decrease in crop growth, yield, and overall agricultural production worldwide [3]. In high-salinity conditions, cells expel Na^+^ ions and produce osmoprotectants, changing their photosynthetic and antioxidant levels [4]. High salt levels in the soil can disturb the balance of ions in plants, hindering water uptake and causing oxidative damage, leading to lower crop yields and reduced crop quality [5]. When plants are under salinity stress, their cells gather reactive oxygen species (ROS), leading to ionic, osmotic, and nutritional stress. The most common ROS produced by plant cells are superoxide anion radicals (O_2_^•−^), hydrogen peroxide (H_2_O_2_), and hydroxyl radicals (OH^•^). This problem is expected to worsen due to high-salinity irrigation water due to low rainfall and inadequate agricultural practices, especially in dry regions where the evapotranspiration exceeds the precipitation [6]. However, adopting sustainable agricultural practices offers hope in mitigating this issue. Developing and implementing these sustainable agricultural practices to mitigate salinity stress’s harmful impact on agricultural production is crucial and necessary. It is the key to meeting the growing food demand and feeding the increasing global population [5]. Plants have developed defense mechanisms to cope with harmful ROS from toxic metals by activating the antioxidant defense system. Salt stress increases antioxidant enzymes in plants, such as superoxide dismutase (SOD), peroxidase (POD), catalase (CAT), glutathione reductase (GR), and ascorbate peroxidase (APx). Long-term exposure to salt stress leads to enhanced antioxidant activity that indicates the scavenging of H_2_O_2_ [7]. There have been reports of the applications of different phytohormones used to reduce salt stress, such as strigolactones [8,9,10], gibberellin [11], salicylic acid [12], and ascorbic acid [13,14]. The harmful effects of saline soils on crops have led to the use of expensive technologies, which can also harm the ecosystem. Therefore, there is a need to develop cost-effective, eco-friendly, and practical solutions. Currently, nanoparticles (NPs) are widely used and are significant contributors across the globe as plant growth regulators [15]. As tiny, unique entities, their properties improve plants’ growth and performance when faced with different stress factors, such as salt stress [16]. Lashkary et al. [17] reported the application of NPs for inducing oxidative stress in numerous plants.

Titanium dioxide (TiO_2_) NPs are some of the most commonly used nanoparticles to address environmental challenges, improving numerous plants’ biochemical and physiological properties [18]. Lei et al. [19] reported improvements in antioxidant enzyme activity and photosynthetic rate with the application of TiO_2_, leading to enhanced crop growth. The enhanced antioxidant enzyme activity reduces plants’ H_2_O_2_ and MDA production, thus improving their proline content and plant growth [20]. Another study found that exogenously applied TiO_2_-NPs improved salt stress in maize crops by enhancing their chlorophyll contents, phenolics, and yield [21]. Khan [22] discovered that applying TiO_2_-NPs can mitigate tomato salt stress, improving the chlorophyll content, enzyme activities, and yield. TiO_2_ acts as a plant stimulant, activating defense mechanisms against different stress factors [23]. However, several reports have indicated that excess nanoparticle application can cause toxic effects when dosed in high concentrations [24]. These effects have been reported to vary with the environment and plant species and the applied concentration. As per Gohari et al. [24], TiO_2_-NPs at the rate of 200 ppm should lead to significant improvements in Moldavian plants against salinity. Similarly, Amooghaie et al. [25] studied the application of 80 ppm TiO_2_ NPs against *Carum copticum*. High concentrations of TiO_2_ in plants have been reported to cause toxic effects, resulting in stunted growth. Rico et al. [26] reported that TiO_2_ concentration leads to higher ROS production, chlorophyll degradation, and increased cellular toxicity. Therefore, reducing the concentration of TiO_2_ is essential [27].

One of these vegetables is eggplant (*Solanum melongena* L.), which belongs to the Solanaceous family and is cultivated worldwide [28]. Eggplant is a plant that is sensitive to salt stress. When exposed to high salt levels, its growth and yield decrease [29]. However, different varieties of eggplant show varying responses to different types of stress. This variation is due to differences in their physical, biochemical, and physiological traits [30]. Eggplants contain high levels of phenolic acids, which have numerous health benefits. This has led to research on the salt tolerance of eggplants growing in harsh, high-salinity conditions [31]. TiO_2_-NP foliar application has been used on eggplant against fungus (*Phomopsis vexans*), bacteria (*Ralstonia solanacearum*), and root-rot nematode (*Meloidogyne incognita*) at 200 ppm, and significant changes were observed [32]. So, we aim to explore the effects TiO_2_-NPs on the growth, physiological responses, and overall health of eggplant (*Solanum melongena*) seedlings when subjected to moderate and high salinity stress by assessing various growth parameters, photosynthetic efficiency, toxic ion content, stress indicators, and antioxidative enzymatic activity. This research seeks to explain the possible influence of TiO_2_ nanoparticles in lessening or intensifying the negative consequences of salinity stress on eggplant seedlings.

## 2. Materials and Methods

### 2.1. Characterization of TiO_2_-NPs

The solid TiO_2_-NPs were purchased from Sigma-Aldrich (St. Louis, MO, USA). Field emission scanning electron microscopy (FE-SEM-Nova Nano-450, FEI, USA) was used for morphological analysis of the TiO_2_ nanoparticles. X-ray diffraction (XRD-PANalytical Empyrean, Malvern, Almelo, The Netherlands) was used for analysis with a scanning angle in the range of 0° ≤ 2θ ≤ 90° at a scanning rate of 2°/min to study the structure and phase purity of the TiO_2_ nanoparticles. Fourier transform infrared spectroscopy (FTIR-PerkinElmer, Waltham, MA, USA) was utilized to analyze the TiO_2_ particles in the 5000–500 cm^−1^ range. Thermogravimetric analysis (TGA-pyris 4000 PerkinElmer, Waltham, MA, USA) was used to study the thermal stability of the TiO_2_ nanoparticles.

### 2.2. Plant Growth and Experimental Conditions

**Figure 1:**
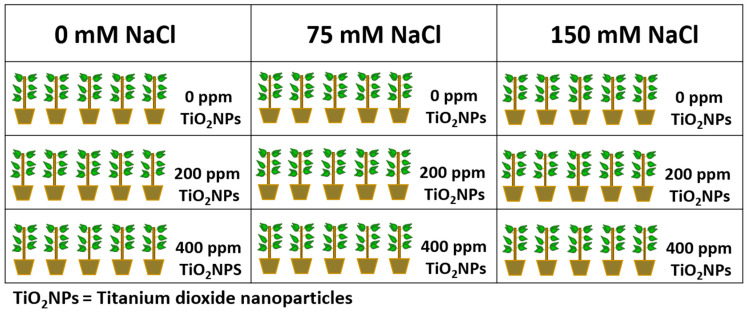
Experimental layout of one replication for eggplant seedlings.

Plants were obtained from Agrico-Qatar when they were two weeks old. The seedlings were placed in a greenhouse facility at Qatar University. After two weeks of acclimatization, the uniformly sized seedlings were transplanted into peat moss pots (Ø 20 cm) (one plant in each pot). Before the experiment, plants were only irrigated with a Hoagland nutrient solution [33] for one week to maintain growth and prevent stress. To obtain precise results from our experiments, healthy plants were selected after one week to perform the experiments. The experiment was performed in the greenhouse facility at Qatar University with a mean daytime temperature of 28 °C and nighttime temperature of 14 °C. The relative humidity varied between 60% and 80%, and the amount of light (PAR) ranged from 300 to 500 μmols/m^2^/s. A completely randomized design was used with two factors, i.e., nanoparticles and salt stress. The seedlings were subjected to three levels of salinity (0 mM, 75 mM, and 150 mM) through irrigation with the Hoagland solution by using sodium chloride (NaCl) powder (99% Sigma Aldrich). Furthermore, the exogenous foliar application of titanium dioxide (TiO_2_) nanoparticles (NPs) was applied at the rates of 0 ppm, 200 ppm, and 400 ppm twice a week. The titanium (IV) oxide (TiO_2_, 99.7%) trace nanoparticles were purchased from Sigma-Aldrich, and later, dispersed solutions of 200 and 400 ppm were prepared by mixing them into deionized water followed by ultra-sonication treatment to prepare a homogenous and well-dispersed TiO_2_ solution. The experiment had three replications and five pots in each replication (Figure 1).

### 2.3. Chlorophyll Fluorescence

The chlorophyll fluorescence and nonphotochemical quenching (NPQ) of eggplant leaves were measured at the end of the trial using the FluorPen (FP-100, Photon Systems Instruments, Drásov, Czech Republic). Fully expanded leaves of eggplant plants were used for Fv′/Fm′ in the daytime. For Fv/Fm and NPQ, leaves were exposed to dark conditions for 1 h using light-retaining clips, and then, readings were taken [34].

### 2.4. Growth Attributes

The relative chlorophyll content was measured using a SPAD meter (SPAD-502 plus Konica Minolta, Tokyo, Japan). After harvesting the eggplant seedlings, the number of leaves, the length of shoots, the length of roots, and the fresh biomass of the plants were measured. Dry biomass was then generated by placing the harvested plants in a hot air oven at 65 °C for five days.

### 2.5. Enzymatic Activity and TSP

The freshly harvested leaves were crushed in liquid nitrogen and stored at −80 °C to measure the enzymatic activity and for further analysis. To measure SOD (1.15.1.1), POD (1.11.1.7), and CAT (1.11.1.6) activity, 300 mg of powdered leaves was homogenized in chilled conditions with 3 mL sodium phosphate buffer (pH 7.8) and centrifuged at 15,000 rpm at 4 °C for 5 min [34]. For SOD activity, 1 mL reaction solution contained 75 mM ethylenediamine tetra-acetic acid, 50 mM nitroblue tetrazolium, 50 mM sodium phosphate buffer, 1.3 µM riboflavin, and 13 mM methionine, and the absorbance was measured at 560 nm using a spectrophotometer [35]. Change and Maehly’s [36] method was used to estimate the POD and CAT activity. The reaction solution of CAT contained 5.9 mM hydrogen peroxide and 50 mM sodium phosphate buffer. In comparison, the POD activity reaction solution consisted of 20 mM guaiacol, 50 mM sodium phosphate buffer, and 40 mM hydrogen peroxide. The absorbance was read at 240 and 470 nm.

The APx (1.11.1.11) activity was measured using [37]. The reaction solution for APx contained 50 mM sodium phosphate buffer, 0.5 mM ascorbate, 0.1 mM ethylenediamine tetra-acetic acid, and 1.2 mM hydrogen peroxide, and absorbance was measured at 290 nm. For GR (1.6.4.2) activity, the reaction solution contained 100 mM potassium phosphate buffer, 0.5 mM glutathione oxidase, 2 mM ethylenediamine tetra-acetic acid, and 0.2 mM nicotinamide adenine dinucleotide phosphate hydrogen, and the absorbance was measured at 340 nm [38]. To measure the total soluble protein (TSP) content, 0.5 g of fresh leaves was homogenized with 1 mL phosphate saline buffer (pH 7.2) at 10,000 rpm for 5 min. The extracted solution was then added to deionized water and Coomassie blue dye. The absorbance was measured at 595 nm [39] using a UV/Vis spectrophotometer, Lambda-25 (Perkins Elmer, Waltham, MA, USA).

### 2.6. Stress Indicators and Toxic Ion

For stress indicators, H_2_O_2_ and MDA were measured. For H_2_O_2_ and MDA estimation, 300 mg of crushed leaves was taken and homogenized in 3 mL of 0.1% trichloroacetic acid and centrifuged at 12,000 rpm for 15 min. For H_2_O_2_, the reaction solution contained 1 M potassium iodide and 10 mM potassium phosphate buffer, and the absorbance was read at 390 nm [40]. The MDA was carried out by following [41]. The absorbance was measured at 532 and 600 nm using a UV/Vis spectrophotometer, Lambda-25 (Perkins Elmer, Waltham, MA, USA). The reaction solution contained 0.5% thiobarbituric acid and 20% trichloroacetic acid.

To measure the Na^+^ ions in eggplant plants, 0.1 g dried leaves were wet-digested at 350 °C by adding 2 mL sulfuric acid and 0.2 mL hydrogen peroxide to stabilize the phosphorous to obtain a colorless solution [42]. The inductively coupled plasma optical emission spectrometer, (ICP-OES) Optima 5300 DV (Perkins Elmer, Waltham, MA, USA) was used to measure the Na concentration.

### 2.7. Statistical Analysis

The data were analyzed using the Stastix 8.1 (Tallahassee, FL, USA) software package, and an analysis of variance (ANOVA) is shown in Supplementary Table S1. The least significant difference test (LSD) was conducted to compare the means at a probability level of 5% [43]. Pearson’s correlation and principal component analysis (PCA) were constructed using Rstat.

## 3. Results

Under salt stress conditions, the eggplant seedling leaves exhibited necrotic symptoms, alleviated by applying TiO_2_-NPs to the foliage (Supplementary Figure S1).

### 3.1. Characterization of Nanoparticles

The characterization of TiO_2_-NPs was confirmed before their application by XRD, FTIR, TGA, and SEM.

#### 3.1.1. XRD and FTIR Analysis

**Figure 2:**
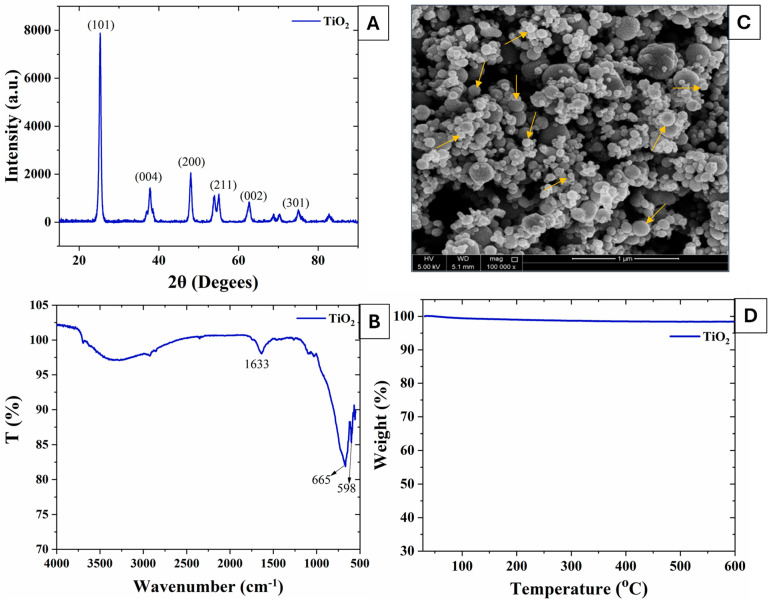
(**A**) XRD; (**B**) FTIR; (**C**) SEM; (**D**) TGA of TiO_2_-NPs. Arrows in (**C**) indicate the size distribution of TiO_2_-NPs.

Structural analysis of the TiO_2_ was carried out using an XRD analysis (Figure 2A), where the pure crystalline behavior of the TiO_2_ nanoparticles can be observed, with sharp peaks present. The XRD pattern shows the characteristic peaks at 25.2°, 37.6°, 48°, 53.8°, 55°, 62.4°, 68.8°, and 74.8°, which are in agreement with the (JPCD:01-078-2486), which confirms the anatase pattern of TiO_2_-NPs [44]. The FTIR analysis was conducted and is depicted in Figure 2B. The broad peak at 3400–3500 cm^−1^ in the FTIR spectra of TiO_2_-NPs indicates the hydroxyl group O-H due to the moisture presence of TiO_2_ [45]. The Ti-OH stretching vibration can also be observed at 1633 cm^−1^ of the TiO_2_ spectra. Furthermore, the distinct peaks of Ti-O-Ti vibrations were noted at wavenumbers below 1000 cm^−1^ [46].

#### 3.1.2. SEM and TGA

Scanning electron microscopy (SEM) images of the TiO_2_ were taken at 100,000× magnification and are depicted in Figure 2C, which shows the spherical morphology of the TiO_2_ nanoparticles. TiO_2_ was distributed homogeneously without agglomeration, and the particle size range was observed to be from 30 to 50 nm. A few nanoparticles above 50 nm in size were also observed. However, most of the nanoparticles were smaller than 50 nm in size.

The results depicted in Figure 2D demonstrate that the TiO_2_ sample maintained its stability without any notable weight loss, confirming its pure composition. A minor total weight loss of around 1.4% was observed in the temperature range of 600 °C due to some moisture removal. So, the TGA of titania (TiO_2_) shows that it is a very thermally and chemically stable compound [47].

### 3.2. Chlorophyll Fluorescence

**Figure 3:**
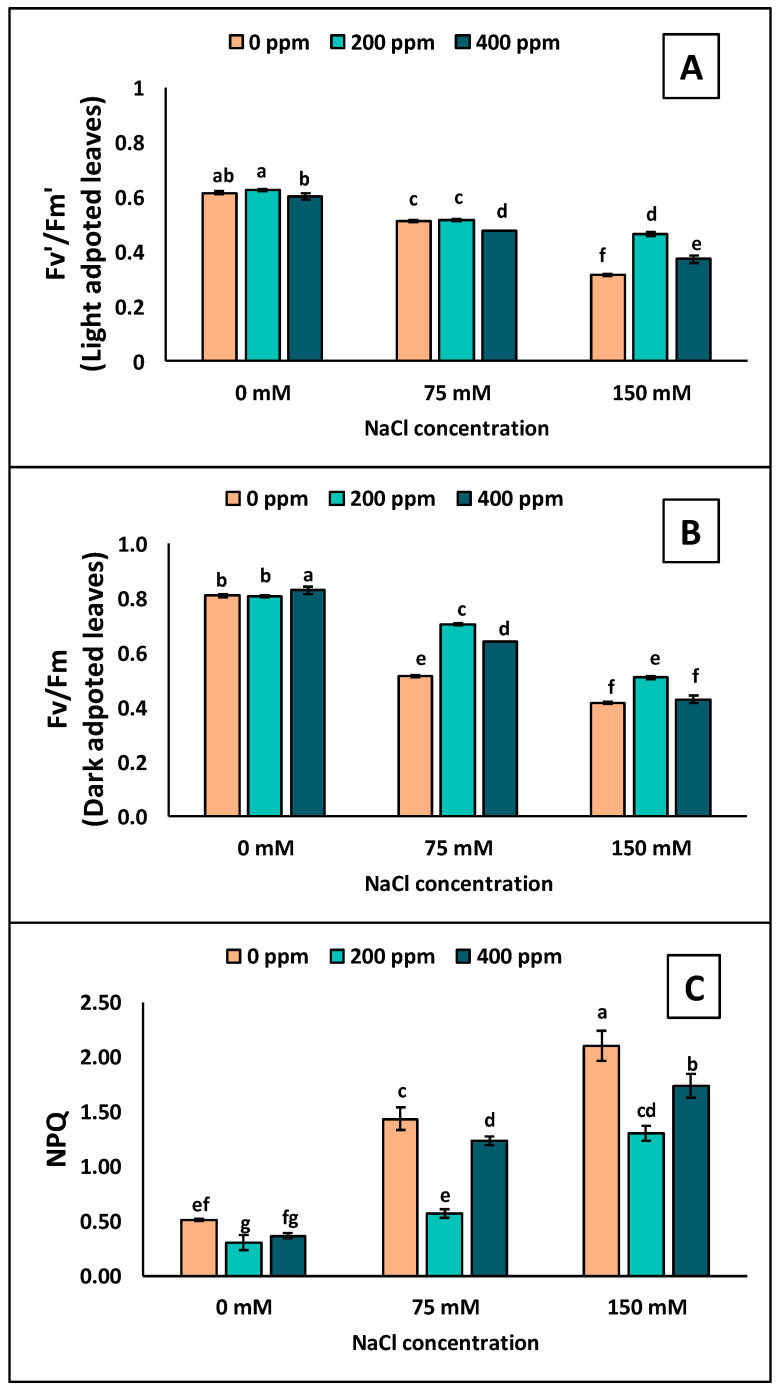
Measurements of the chlorophyll fluorescence in the leaves of eggplant seedlings grown under different levels of salinity stress (0, 75, 150 mM) with TiO_2_-NP application (0, 200, 400 ppm). (**A**) Fv′/Fm′; (**B**) Fv/Fm; (**C**) NPQ. Lettering showing the statistical differences according to LSD test. Values are mean ±S.E. at *p* < 0.05.

The chlorophyll attributes of eggplant seedlings were significantly affected by varying levels of salt stress when TiO_2_-NPs were applied (Supplementary Table S1, Figure 3). The eggplant seedling leaves exhibited significantly reduced Fv′/Fm′ and Fv/Fm when exposed to salt stress conditions. The decrease was more significant at 150 mM than at 0 and 75 mM NaCl. The seedlings treated with different concentrations of TiO_2_-NPs displayed smaller reductions in Fv′/Fm′ and Fv/Fm. At 75 mM NaCl, the seedlings treated with 200 ppm of TiO_2_-NPs showed no significant difference in Fv′/Fm′ value compared with 0 mM (Figure 3A); instead, the higher concentration (400 ppm) of TiO_2_-NPs showed a larger decrease in Fv′/Fm′ at 75 mM. However, at 150 mM, both TiO_2_-NP concentrations showed higher Fv′/Fm′ values than 0 mM. Moreover, the TiO_2_-NPs at 200 ppm showed less decline than 400 ppm under 150 mM salt stress condition (Figure 3A).

Similarly, 200 and 400 ppm TiO_2_-NPs exhibited smaller decreases in Fv/Fm than 75 mM of NaCl compared to 0 mM (Figure 3B). At 150 mM NaCl stress, 400 ppm showed no significant difference compared to 0 mM. The NPQ increased with increasing salt levels. The maximum NPQ was observed in eggplant seedling leaves at 150 mM with no TiO_2_-NP application (Figure 3C). The application of TiO_2_-NPs at 200 and 400 ppm showed smaller increases at 75 and 150 mM of NaCl, while 200 ppm of TiO_2_-NPs showed the minimum increase in NPQ (Figure 3C).

### 3.3. Growth Attributes

**Figure 4:**
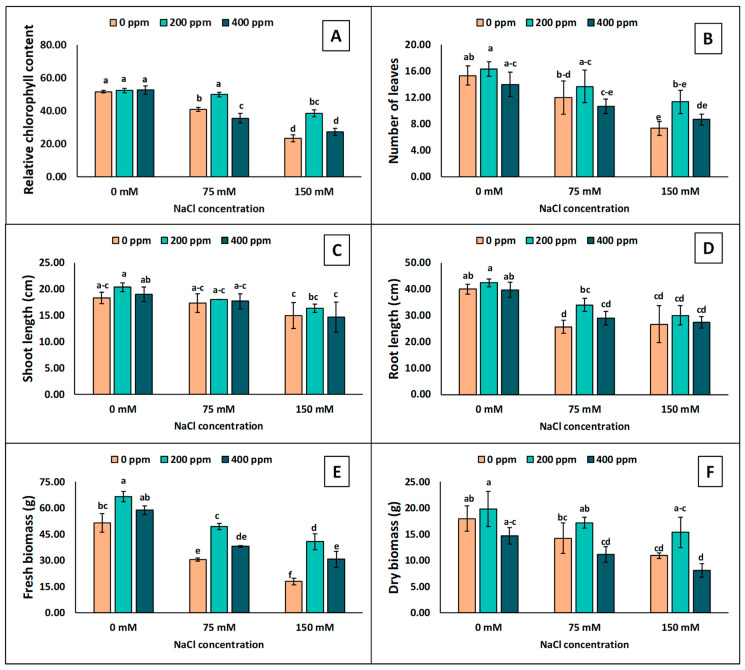
Measurements of the growth attributes in the leaves of eggplant seedlings grown under different levels of salinity stress (0, 75, 150 mM) with TiO_2_-NP application (0, 200, 400 ppm). (**A**) Relative chlorophyll content; (**B**) number of leaves; (**C**) shoot length; (**D**) root length; (**E**) fresh biomass; (**F**) dry biomass. Lettering showing the statistical differences according to LSD test. Values are mean ±S.E. at *p* < 0.05.

The eggplant seedlings’ growth attributes were significantly affected by salinity with TiO_2_-NPs (Figure 4). Leaf greenness declined by increasing the salinity level (Figure 4A). The minimum decline was observed at 75 mM NaCl with 200 ppm TiO_2_-NPs. Similarly, at 150 mM of NaCl, 200 ppm TiO_2_-NPs showed a smaller decline in leaf greenness than 0 and 150 mM of NaCl (Figure 4A). The maximum decline was observed at 75 mM of NaCl without TiO_2_-NP application, while at 150 mM NaCl with 0, and 400 ppm TiO_2_-NPs, there was a maximum decrease. The growth attributes of eggplant seedlings were decreased by increasing the salinity levels (Figure 4B–D). A higher decrease was observed at 150 mM than at 75 mM NaCl. TiO_2_-NP application at 200 ppm showed a smaller decrease than 0 and 400 ppm, but the ANOVA showed that the difference was statistically non-significant (Supplementary Table S1). Under salt stress, fresh biomass and dry biomass also showed similar results in eggplant seedlings (Figure 4E,F). The decrease in fresh biomass was greater at 150 mM NaCl with 0 ppm TiO_2_-NP application. The minimum dry biomass was measured at 0 and 400 ppm under 150 mM NaCl. Meanwhile, 200 ppm TiO_2_-NPs exhibited a smaller decrement than 400 ppm TiO_2_-NPs at 75 and 150 mM NaCl.

### 3.4. Enzymatic Activity and TSP

**Figure 5:**
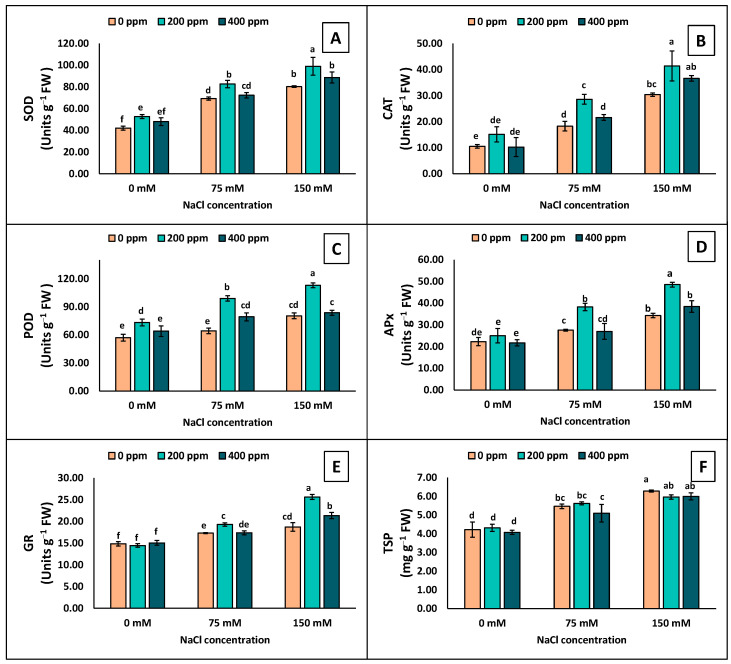
Measurements of the antioxidative enzymes in the leaves of eggplant seedlings grown under different levels of salinity stress (0, 75, 150 mM) with TiO_2_-NP application (0, 200, 400 ppm). (**A**) SOD; (**B**) CAT; (**C**) POD; (**D**) APx; (**E**) GR; (**F**) TSP. Lettering showing the statistical differences according to LSD test. Values are mean ±S.E. at *p* < 0.05.

The antioxidative enzyme activity and TSP were significantly increased by increasing salt stress by spraying with TiO_2_-NPs (Figure 5). APx, CAT, SOD, POD, and GR showed higher activity when exposed to salt stress conditions. The maximum SOD, POD, CAT, APx, and GR activity was detected at 150 mM NaCl with 200 ppm foliar TiO_2_-NP application (Figure 5). Meanwhile, the minimum activity was observed with 0 ppm and 400 ppm TiO_2_-NPs at 75 and 150 mM NaCl. The 200 ppm TiO_2_-NPs significantly increased the antioxidative enzyme activity at 0, 75, and 150 mM NaCl (Figure 5). However, the TSP content increased at 150 mM, while no significant difference was observed with TiO_2_-NPs (Figure 5F).

### 3.5. Stress Indicators and Toxic Ions

**Figure 6:**
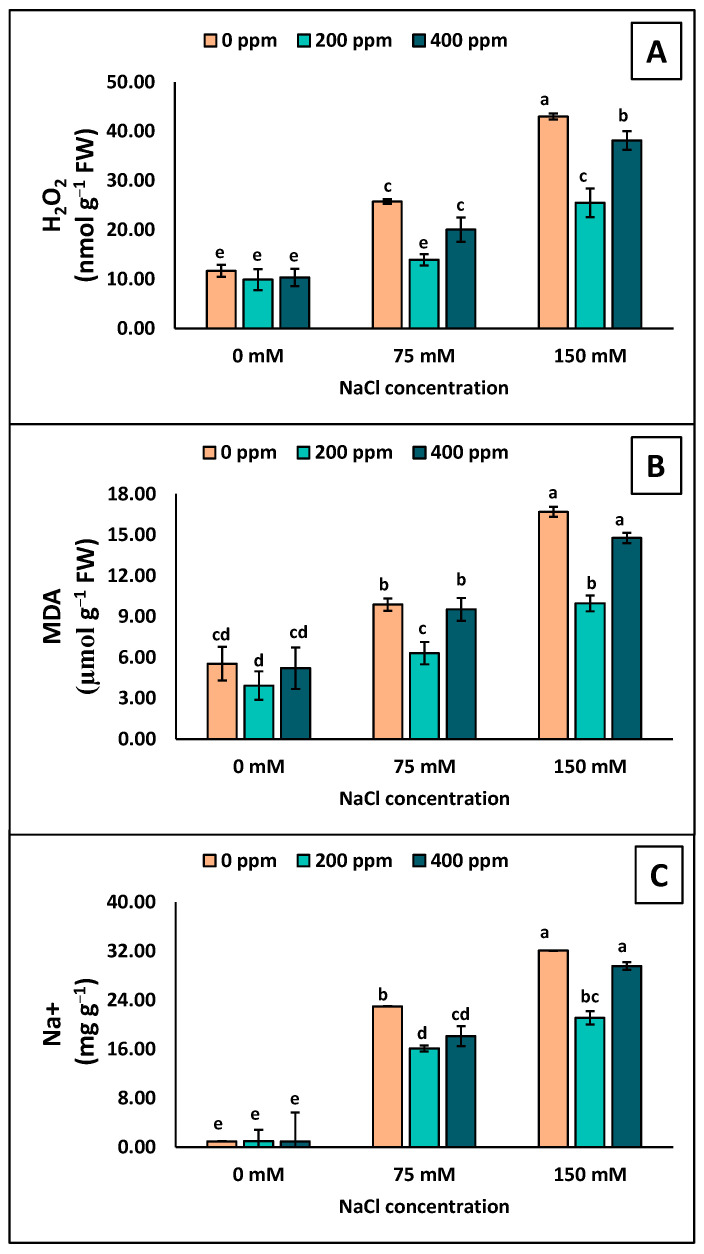
Measurements of the stress indicators and toxic ions in the leaves of eggplant seedlings grown under different levels of salinity stress (0, 75, 150 mM) with TiO_2_-NP application (0, 200, 400 ppm). (**A**) H_2_O_2_; (**B**) MDA; (**C**) Na^+^. Lettering showing the statistical differences according to LSD test. Values are mean ±S.E. at *p* < 0.05.

The stress indicators in the leaves of eggplant seedlings were observed to be higher by increasing the salinity stress (Figure 6A,B). The maximum H_2_O_2_ and MDA contents were observed at 150 mM NaCl with no application of TiO_2_-NPs. The spraying of TiO_2_-NPs on leaves at 200 ppm showed significantly lower H_2_O_2_ and MDA contents than 0 and 400 ppm TiO_2_-NPs (Figure 6A,B). The only significant difference was at 400 ppm TiO_2_-NPs and 150 mM H_2_O_2_ content.

Under salinity stress, the Na ion showed an increment in the leaves of eggplant seedlings by increasing its NaCl concentration (Figure 6C). The maximum Na^+^ was observed in the leaves exposed to 150 mM NaCl. No statistically significant difference was observed at 75 mM NaCl with foliar application of TiO_2_-NPs. Meanwhile, at 150 mM, 200 ppm showed less Na^+^ accumulation in the leaves of eggplant seedlings (Figure 6C).

### 3.6. Pearson’s Correlation

**Figure 7:**
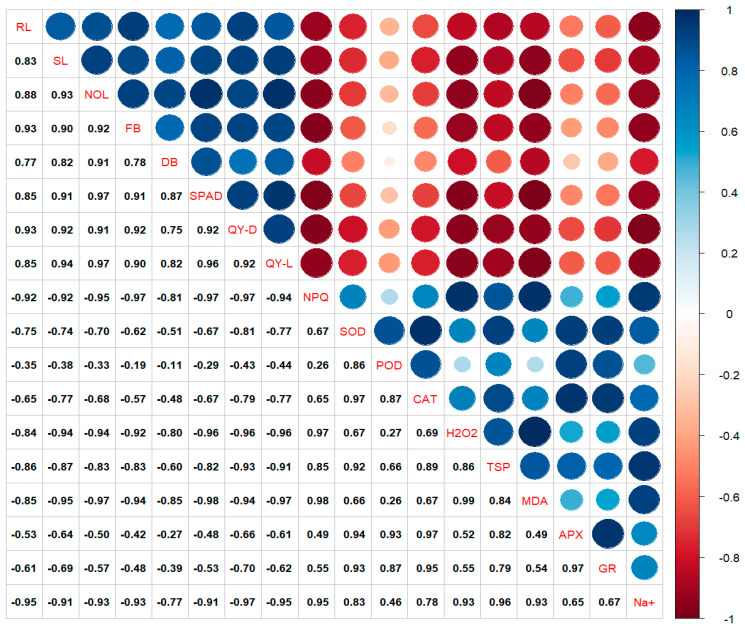
The Pearson’s correlation matrix of exogenous application of TiO_2_-NPs on eggplant seedlings grown under salt toxicity. RL = root length; SL = shoot length; NOL = no. of leaves; FB = fresh biomass; DB = dry Biomass; SPAD = relative chlorophyll content; QY-D = Fv/Fm; QY-L; Fv′/Fm′; NPQ = non photochemical quenching; SOD = superoxide dismutase; POD = peroxidase; CAT = catalase; TSP = total soluble proteins; MDA = malondialdehyde; APX = ascorbate peroxidase; GR = glutathione reductase.

Leaf greenness was significantly and positively correlated with the number of leaves, Fv/Fm, Fv′/Fm′, and fresh biomass and was negatively correlated with NPQ and H_2_O_2_. MDA and H_2_O_2_ were negatively correlated with the shoot length, number of leaves, fresh biomass, Fv/Fm, and Fv′/Fm′, but positively correlated with NPQ (Figure 7). SOD was positively correlated with CAT, APx, GR, and TSP but negative with physiological attributes. Na^+^ ions showed a significant positive correlation with MDA, TSP, H_2_O_2_, and NPQ. They also showed a negative correlation with the root length, shoot length, number of leaves, fresh biomass, leaf greenness, Fv/Fm, and Fv′/Fm′ (Figure 7).

### 3.7. PCA

**Figure 8:**
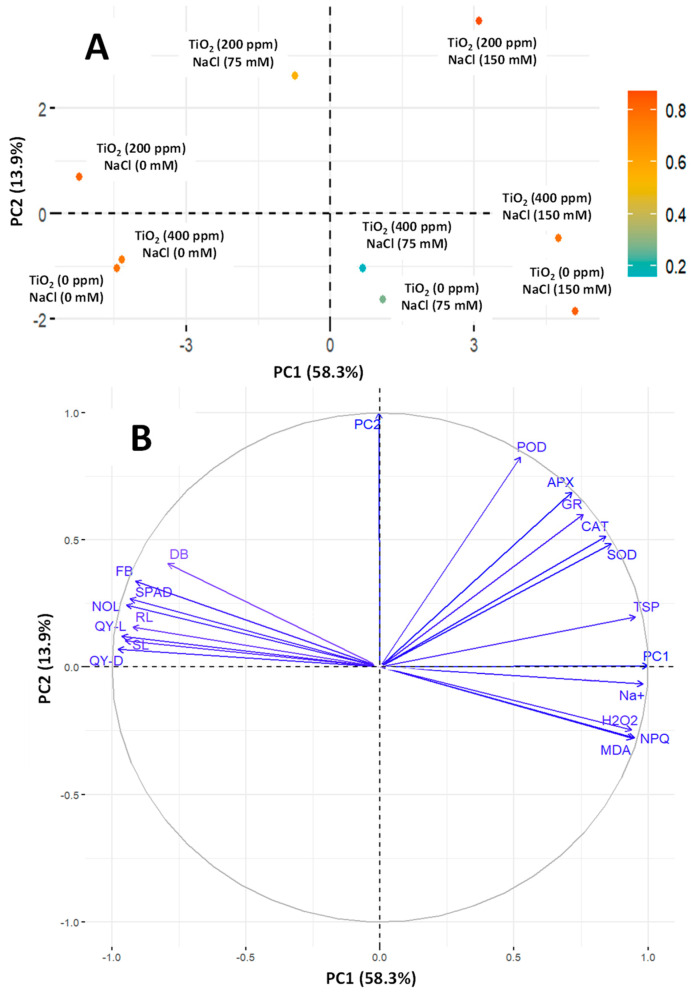
PCA representing eggplant seedlings grown under different levels of salinity stress (0, 75, 150 mM) with TiO_2_-NP application (0, 200, 400 ppm). Contribution of each attribute on axes (**A**) and studied attributes (physiological and biochemical) on axes (**B**).

A PCA was conducted for different investigated variables of eggplant seedlings under salt toxicity by applying TiO_2_-NPs. The analysis depicted the separation due to foliar application of TiO_2_-NPs and salinity stress. PC1 separated the salinity levels, while PC2 separated TiO_2_-NPs. A variation of 58.3% was observed, while PC2 showed 13.9% variability (Figure 8A). A variation of 58.3% was observed in PC1, and NPQ, SOD, CAT, H_2_O_2_, MDA, TSP, GR, and Na^+^ contributed positively, while all growth attributes contributed negatively (Supplementary Table S2). Eighteen variable groups over four sections were explored, which showed negative and positive factor spaces in 2D (Figure 8B). POD, CAT, APx, GR, SOD, and TSP are positioned on the positive section of both axes (X and Y), showing positive associations. The remaining twelve attributes were negatively associated with at least one parameter, as they were positioned on the negative axis.

## 4. Discussion

Agricultural industries are plagued by salinity, one of the oldest and most concerning challenges. Salty conditions significantly impact plant productivity and growth, disrupting various physiological and biochemical processes. Physiological and biochemical processes in a saline environment are affected by the accumulation of Na^+^, which affects nutrient and ion balances [48,49]. Salinity stress affects photosynthesis. NaCl stress disrupts photosystem II (PSII) due to its toxicity to Na^+^, the most sensitive photochemical process [15,50]. Our results indicated that Fv′/Fm′ and Fv/Fm showed a decline under salt stress conditions. Plants face environmental stress when they encounter challenging conditions, as the Fv′/Fm′ and Fv/Fm of PSII decline. This implies that when exposed to environmental stress, the plant suffers from photoinhibition due to excess photon flux density inside the plant [51]. An increase in NPQ is mainly caused by releasing extra energy that can cause damage. This increase is linked to a negative relationship with PSII (Figure 7). It is possible that this could rival photochemical quenching and decrease the number of electrons engaged in photosynthesis, as demonstrated by a decrease in Fv′/Fm′ [15,34]. PSII and NPQ mainly affected plants under stress conditions due to electron acceptor concentrations (NADP^+^ is on the acceptor side of PSI) [52]. Wu et al. [53] and AlKhatib et al. [54] reported results that are in alignment with our results, showing a decrease in Fv′/Fm′ and Fv/Fm and NPQ of eggplant seedlings under salinity stress. At the same time, the TiO_2_-NPs limit the decrease in PSII, which makes the plant tolerate salinity (Figure 3). The foliar application of NPs on plants can synthesize more complexes for light harvesting, increasing photosynthesis and light absorption and leading to tolerance against salinity [16,55,56].

The decrease in photosynthetic attributes was also led by the plants’ chlorophyll content, estimated by leaf greenness. The decline in leaf greenness also decreases plant growth, as illustrated in Figure 7 and Figure 8. The leaf greenness is significant and positively correlated with plant growth attributes. Our results indicated that leaf greenness was positively correlated with PSII and negatively correlated with Na^+^. Increasing the salinity level in plants decreased their growth and leaf greenness (Figure 4). Our results also depicted that applying TiO_2_-NPs under normal conditions enhanced the growth and development of the plants, maintained their growth, and showed smaller decreases under salinity. Previous studies also showed increased the growth and agronomic traits of different plant species exposed to NPs under salinity [24,57,58]. Due to their small size and large surface area, NPs penetrate plant cells easily and alleviate the adverse effects of salinity relatively quickly by improving plant growth. The enhanced growth characteristics of plants might be explained by increased water absorption and less leaf transpiration, which results in improved water relations [59,60,61].

When plants undergo salinity stress, they enhance the production of ROS, i.e., O_2_^•−^, H_2_O_2_, and OH^•^ [16]. Plants that are better at disposing of reactive oxygen species (ROS) are more resistant to oxidative damage than those with weaker ROS removal abilities [56,58]. Exposure to salinity leads to higher ROS production in plants, causing damage to nucleic acids, membrane integrity, cellular metabolism, and various metabolic processes in chloroplasts and mitochondria, inevitably leading to cell death [62]. Our results suggested that eggplant seedlings exposed to salinity showed more accumulation of H_2_O_2_ and MDA (Figure 6). Our correlation and PCA also showed a significant and positive relation with Na^+^ ions, which demonstrated that by increasing the Na^+^ concentration in plants, H_2_O_2_ and MDA also increased (Figure 7 and Figure 8). However, applying TiO_2_-NPs significantly decreases the production of H_2_O_2_ and MDA. Similar findings that NPs decreased the accumulation of H_2_O_2_ and MDA in Zea mays [63], grapes [64], and Moldavin Balm [58] were observed. Plants have a mechanism to maintain the equilibrium in the production and expulsion of ROS. To keep the balance in response to the overproduction of ROS in saline conditions, plants enhance the production of several scavenging antioxidative enzymes. It was convincingly reported that SOD is the primary enzyme that activates and converts the O_2_^•−^ to H_2_O_2_, which CAT and APx further detoxify. The scavenging of O_2_^•−^ by SOD reduced the lipid peroxidation [62]. H_2_O_2_ is also scavenged by POD in the chloroplast [65]. Similarly, the GR enzyme plays a vital role in the ascorbate–glutathione cycle. GR catalyzes the NADPH-dependent reduction in oxidized glutathione, helping protect many plants from oxidative damage induced by salt stress [66]. Our study also observed increased antioxidative enzymes when exposed to salinity stress (Figure 5). However, an increase was observed in the seedlings treated with foliar application of TiO_2_-NPs. Similar findings that TiO_2_-NPs enhanced the production of antioxidative enzymes under salinity stress were observed by Shah et al. [63] and Gohari et al. [24]. Omar et al. [67] reported that applying TiO_2_-NPs under salinity stress on Vicia faba seedlings increased antioxidative enzymes and heat shock proteins (HSPs). TiO_2_-NPs, which are up-regulated HSPs (HSP17.9 and HSP70), act as molecular chaperones and prevent misfolded proteins from aggregating, and their application can therefore protect cells and support the proper folding of newly synthesized proteins [68]. However, the increase in TiO_2_-NPs also caused plant toxicity [24]. The higher concentration of NPs interacts with DNA and damages the root meristem [69]. A higher concentration of NPs increases the ROS, which damage lipids and proteins [70]. In our findings, we also observed that a higher concentration of TiO_2_-NPs (400 ppm) led to lower antioxidative enzymes and higher stress indicators (H_2_O_2_, MDA) than 200 ppm of TiO_2_-NPs. The Na^+^ accumulation in the leaves was also limited following foliar application of TiO_2_-NPs in eggplant under 75 and 150 mM NaCl (Figure 6C). Less accumulation of Na^+^ was shown with higher concentrations of TiO_2_-NPs (400 ppm) at 75 and 150 mM of NaCl, but it did not significantly differ from the control. The Na^+^ accumulation was also found to be positive and significantly correlated with H_2_O_2_ and MDA, which indicated that by increasing the Na^+^ accumulation in leaves, the plant stress indicators also increased (Figure 7). However, Na^+^ is negatively correlated with PSII variables, leaf greenness, the no. of leaves, etc., which demonstrated that all growth and physiological attributes showed decrements by increasing the toxic ion concentration in the plant.

## 5. Conclusions

**Figure 9:**
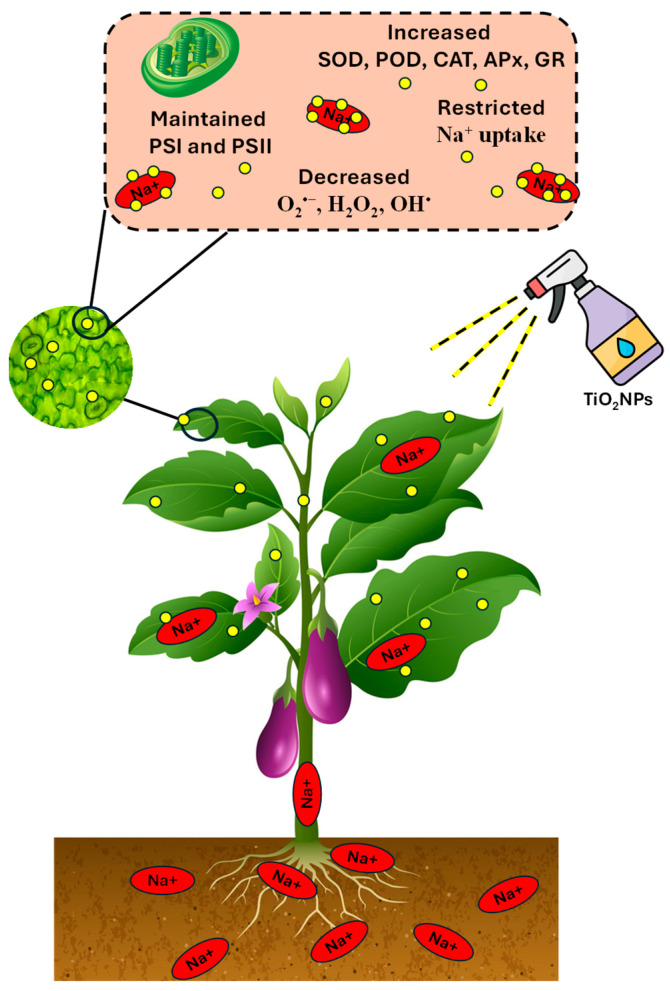
Schematic diagram showing how TiO_2_-NPs mitigate the salt toxicity in eggplant plants.

Moderate (75 mM) and high (150 mM) salt stress caused a significant decrease in the growth and development of the eggplant seedlings (Figure 9). The damage due to salinity was reduced by the application of TiO_2_-NPs in eggplant seedlings by activating antioxidative enzymes and restricting the toxic ion accumulation in leaves. The concentration of 200 ppm of TiO_2_-NPs significantly reduced the salinity effect in plants and improved their growth (shoot and root) and fresh and dry biomass. PSII was maintained in the plants that were treated with 200 ppm TiO_2_-NPs. The application increased the oxidative enzyme activity (SOD, POD, and CAT) and simultaneously limited H_2_O_2_ and MDA accumulation. At a concentration of 400 ppm, high levels of TiO_2_-NPs did not result in any notable variances in the enzymatic activity and growth of the plants compared to the control.

## Data Availability

All data are available within the article.

## Supplementary Materials

The following supporting information can be downloaded at https://www.mdpi.com/article/10.3390/antiox13101209/s1: Figure S1: Effect of TiO_2_NPs on eggplant seedlings at 150 mM NaCl; Table S1: Analysis of Variance for eggplants seedlings under salinity stress with foliar application of TiO_2_-NPs; Table S2: Principal Components for physiological and biochemical responses in the leaves of eggplant seedlings under salinity with application of TiO_2_-NPs seedlings under salinity.

## References

[1] Karaca C., Aslan G.E., Buyuktas D., Kurunc A., Bastug R., Navarro A. (2022). Effects of salinity stress on drip-irrigated tomatoes grown under mediterranean-type greenhouse conditions. Agronomy.

[2] Ahmed M.Z., Gul B., Khan M.A., Watanabe K.N. (2016). Characterization and function of sodium exchanger genes in Aeluropus lagopoides under NaCl stress. Halophytes for Food Security in Dry Lands.

[3] Daba A.W., Qureshi A.S. (2021). Review of soil salinity and sodicity challenges to crop production in the lowland irrigated areas of Ethiopia and its management strategies. Land.

[4] Balasubramaniam T., Shen G., Esmaeili N., Zhang H. (2023). Plants’ response mechanisms to salinity stress. Plants.

[5] Junedi M.A., Mukhopadhyay R., Manjari K.S. (2023). Alleviating salinity stress in crop plants using new engineered nanoparticles (ENPs). Plant Stress.

[6] Amer R. (2021). Spatial relationship between irrigation water salinity, waterlogging, and cropland degradation in the arid and semi-arid environments. Remote Sens..

[7] Khalid M.F., Abou Elezz A., Jawaid M.Z., Ahmed T. (2023). Salicylic acid restricts mercury translocation by activating strong antioxidant defense mechanisms in sweet pepper (*Capsicum annum* L.). Environ. Technol. Innov..

[8] Khalid M.F., Shafqat W., Khan R.I., Jawaid M.Z., Hussain S., Saqib M., Rizwan M., Ahmed T. (2024). Unveiling the resilience mechanism: Strigolactones as master regulators of plant responses to abiotic stresses. Plant Stress.

[9] Zhang X., Zhang L., Ma C., Su M., Wang J., Zheng S., Zhang T. (2022). Exogenous strigolactones alleviate the photosynthetic inhibition and oxidative damage of cucumber seedlings under salt stress. Sci. Hortic..

[10] Ling F., Su Q., Jiang H., Cui J., He X., Wu Z., Zhang Z., Liu J., Zhao Y. (2020). Effects of strigolactone on photosynthetic and physiological characteristics in salt-stressed rice seedlings. Sci. Rep..

[11] Liu J., Wu Y., Dong G., Zhu G., Zhou G. (2023). Progress of research on the physiology and molecular regulation of sorghum growth under salt stress by gibberellin. Int. J. Mol. Sci..

[12] Mir R.A., Somasundaram R. (2021). Salicylic acid and salt stress tolerance in plants: A review. J. Stress Physiol. Biochem..

[13] Khan A., Ashraf M. (2008). Exogenously applied ascorbic acid alleviates salt-induced oxidative stress in wheat. Environ. Exp. Bot..

[14] El-Beltagi H.S., Ahmad I., Basit A., Shehata W.F., Hassan U., Shah S.T., Haleema B., Jalal A., Amin R., Khalid M.A. (2022). Ascorbic acid enhances growth and yield of sweet peppers (*Capsicum annum*) by mitigating salinity stress. Gesunde Pflanz..

[15] Mahawar L., Živčák M., Barboricova M., Kovár M., Filaček A., Ferencova J., Vysoká D.M., Brestič M. (2024). Effect of copper oxide and zinc oxide nanoparticles on photosynthesis and physiology of *Raphanus sativus* L. under salinity stress. Plant Physiol. Biochem..

[16] Khalid M.F., Iqbal Khan R., Jawaid M.Z., Shafqat W., Hussain S., Ahmed T., Alina Marc R. (2022). Nanoparticles: The plant saviour under abiotic stresses. Nanomaterials.

[17] Lashkary M., Moghaddam M., Asgharzade A., Tatari M. (2021). Titanium dioxide nanoparticle is involved in mitigating NaCl-induced *Calendula officinalis* L. by activation of antioxidant defense system and accumulation of osmolytes. Plant Physiol. Biochem..

[18] Mustafa N., Raja N.I., Ilyas N., Ikram M., Mashwani Z.U.R., Ehsan M. (2021). Foliar applications of plant-based titanium dioxide nanoparticles to improve agronomic and physiological attributes of wheat (*Triticum aestivum* L.) plants under salinity stress. Green Process. Synth..

[19] Lei Z., Mingyu S., Xiao W., Chao L., Chunxiang Q., Liang C., Fashui H. (2008). Antioxidant stress is promoted by nano-anatase in spinach chloroplasts under UV-B radiation. Biol. Trace Elem. Res..

[20] Abdel Latef A.A.H., Srivastava A.K., El-sadek M.S.A., Kordrostami M., Tran L.S.P. (2018). Titanium dioxide nanoparticles improve growth and enhance tolerance of broad bean plants under saline soil conditions. Land Degrad. Dev..

[21] Hu J., Wu X., Wu F., Chen W., White J.C., Yang Y., Wang X. (2020). Potential application of titanium dioxide nanoparticles to improve the nutritional quality of coriander (*Coriandrum sativum* L.). J. Hazard. Mater..

[22] Khan M.N. (2016). Nano-Titanium Dioxide (nano-TiO_2_) Mitigates NaCl Stress by Enhancing Antioxidative Enzymes and Accumulation of Compatible Solutes in Tomato (Lycopersicon esculentum Mill.).

[23] Mustafa N., Raja N.I., Ilyas N., Abasi F., Ahmad M.S., Ehsan M., Proćków J. (2022). Exogenous application of green titanium dioxide nanoparticles (TiO_2_ NPs) to improve the germination, physiochemical, and yield parameters of wheat plants under salinity stress. Molecules.

[24] Gohari G., Mohammadi A., Akbari A., Panahirad S., Dadpour M.R., Fotopoulos V., Kimura S. (2020). Titanium dioxide nanoparticles (TiO_2_ NPs) promote growth and ameliorate salinity stress effects on the essential oil profile and biochemical attributes of Dracocephalum Moldavia. Sci. Rep..

[25] Amooaghaie R., Majidi M., Farhadian S. (2022). Impact of nano-TiO_2_ on salt stress tolerance of Carum copticum. J. Plant Process Funct..

[26] Rico C.M., Peralta-Videa J.R., Gardea-Torresdey J.L. (2015). Chemistry, biochemistry of nanoparticles, and their role in antioxidant defense system in plants. Nanotechnology and Plant Sciences: Nanoparticles and Their Impact on Plants.

[27] Tan W., Peralta-Videa J.R., Gardea-Torresdey J.L. (2018). Interaction of titanium dioxide nanoparticles with soil components and plants: Current knowledge and future research needs–a critical review. Environ. Sci. Nano.

[28] Jiang Z., Shen L., He J., Du L., Xia X., Zhang L., Yang X. (2023). Functional analysis of SmMYB39 in salt stress tolerance of eggplant (*Solanum melongena* L.). Horticulturae.

[29] Hanachi S., Van Labeke M.C., Mehouachi T. (2014). Application of chlorophyll fluorescence to screen eggplant (*Solanum melongena* L.) cultivars for salt tolerance. Photosynthetica.

[30] Mustafa Z., Ayyub C.M., Amjad M., Ahmad R. (2017). Assessment of biochemical and ionic attributes against salt stress in eggplant (*Solanum melongena* L.) genotypes. J. Anim. Plant Sci..

[31] Brenes M., Solana A., Boscaiu M., Fita A., Vicente O., Calatayud Á., Plazas M. (2020). Physiological and biochemical responses to salt stress in cultivated eggplant (*Solanum melongena* L.) and in *S. insanum* L., a close wild relative. Agronomy.

[32] Khan M., Siddiqui Z.A., Parveen A., Khan A.A., Moon I.S., Alam M. (2022). Elucidating the role of silicon dioxide and titanium dioxide nanoparticles in mitigating the disease of the eggplant caused by Phomopsis vexans, Ralstonia solanacearum, and root-knot nematode Meloidogyne incognita. Nanotechnol. Rev..

[33] Hoagland D.R., Arnon D.I. (1938). The Water-Culture Method for Growing Plants without Soil.

[34] Khalid M.F., Vincent C., Morillon R., Anjum M.A., Ahmad S., Hussain S. (2021). Different strategies lead to a common outcome: Different water-deficit scenarios highlight physiological and biochemical strategies of water-deficit tolerance in diploid versus tetraploid Volkamer lemon. Tree Physiol..

[35] Giannopolitis C.N., Ries S.K. (1977). Superoxide dismutases: I. Occurrence in higher plants. Plant Physiol..

[36] Change B., Maehly A.C. (1955). Assay of catalases and peroxidase. Methods Enzym..

[37] Nakano Y., Asada K. (1981). Hydrogen peroxide is scavenged by ascorbate-specific peroxidase in spinach chloroplasts. Plant Cell Physiol..

[38] Foyer C.H., Halliwell B. (1976). The presence of glutathione and glutathione reductase in chloroplasts: A proposed role in ascorbic acid metabolism. Planta.

[39] Bradford M.M. (1976). A rapid and sensitive method for the quantitation of microgram quantities of protein utilizing the principle of protein-dye binding. Anal. Biochem..

[40] Velikova V., Yordanov I., Edreva A.J.P.S. (2000). Oxidative stress and some antioxidant systems in acid rain-treated bean plants: Protective role of exogenous polyamines. Plant Sci..

[41] Heath R.L., Packer L. (1968). Photoperoxidation in isolated chloroplasts: I. Kinetics and stoichiometry of fatty acid peroxidation. Arch. Biochem. Biophys..

[42] Ryan J., Estefan G., Rashid A. (2001). Soil and Plant Analysis Laboratory Manual.

[43] Gomez K.A., Gomez A.A. (1984). Statistical Procedures for Agricultural Research.

[44] Matouke M.M. (2019). FTIR study of the binary effect of titanium dioxide nanoparticles (nTiO_2_) and copper (Cu^2+^) on the biochemical constituents of liver tissues of catfish (*Clarias gariepinus*). Toxicol. Rep..

[45] Hamdan S.A., Ibrahim I.M., Ali I.M. (2020). Comparison of anatase and rutile TiO_2_ nanostructure for gas sensing application. Dig. J. Nanomater. Biostructures.

[46] Sacco A., Mandrile L., Tay L.L., Itoh N., Raj A., Moure A., Del Campo A., Fernandez J.F., Paton K.R., Wood S. (2023). Quantification of titanium dioxide (TiO_2_) anatase and rutile polymorphs in binary mixtures by Raman spectroscopy: An interlaboratory comparison. Metrologia.

[47] Alsaiari M.A., Alhemiary N.A., Umar A., Hayden B.E. (2020). Growth of amorphous, anatase and rutile phase TiO_2_ thin films on Pt/TiO_2_/SiO_2_/Si (SSTOP) substrate for resistive random access memory (ReRAM) device application. Ceram. Int..

[48] Munns R., Tester M. (2008). Mechanisms of salinity tolerance. Annu. Rev. Plant Biol..

[49] Khalid M.F., Hussain S., Anjum M.A., Ali M.A., Ahmad S., Ejaz S., Ali S., Usman M., Haque E.U., Morillon R. (2020). Efficient compartmentalization and translocation of toxic minerals lead tolerance in volkamer lemon tetraploids more than diploids under moderate and high salt stress. Fruits.

[50] Jajoo A. (2012). Changes in photosystem II in response to salt stress. Ecophysiology and Responses of Plants under Salt Stress.

[51] Bjorkman O. (1987). Photon yield of O_2_ evolution and chlorophyll fluorescence characteristics at 77 K among vascular plants of diverse origins. Planta.

[52] Maxwell K., Johnson G.N. (2000). Chlorophyll fluorescence—A practical guide. J. Exp. Bot..

[53] Wu X., Zhu Z., Li X., Zha D. (2012). Effects of cytokinin on photosynthetic gas exchange, chlorophyll fluorescence parameters and antioxidative system in seedlings of eggplant (*Solanum melongena* L.) under salinity stress. Acta Physiol. Plant..

[54] Alkhatib R., Abdo N., Mheidat M. (2021). Photosynthetic and ultrastructural properties of eggplant (*Solanum melongena*) under salinity stress. Horticulturae.

[55] Ali S., Mehmood A., Khan N. (2021). Uptake, translocation, and consequences of nanomaterials on plant growth and stress adaptation. J. Nanomater..

[56] Li Z., Juneau P., Lian Y., Zhang W., Wang S., Wang C., Shu L., Yan Q., He Z., Xu K. (2020). Effects of titanium dioxide nanoparticles on photosynthetic and antioxidative processes of Scenedesmus obliquus. Plants.

[57] Singh S., Husen A. (2019). Role of nanomaterials in the mitigation of abiotic stress in plants. Nanomaterials and Plant Potential.

[58] Mohammadi M.H.Z., Panahirad S., Navai A., Bahrami M.K., Kulak M., Gohari G. (2021). Cerium oxide nanoparticles (CeO_2_-NPs) improve growth parameters and antioxidant defense system in Moldavian Balm (*Dracocephalum moldavica* L.) under salinity stress. Plant Stress.

[59] Martínez-Ballesta M.C., Zapata L., Chalbi N., Carvajal M. (2016). Multiwalled carbon nanotubes enter broccoli cells enhancing growth and water uptake of plants exposed to salinity. J. Nanobiotechnol..

[60] Alsaeedi A., El-Ramady H., Alshaal T., El-Garawany M., Elhawat N., Al-Otaibi A. (2019). Silica nanoparticles boost growth and productivity of cucumber under water deficit and salinity stresses by balancing nutrients uptake. Plant Physiol. Biochem..

[61] Chen D., Wang S., Yin L., Deng X. (2018). How does silicon mediate plant water uptake and loss under water deficiency?. Front. Plant Sci..

[62] Gill S.S., Tuteja N. (2010). Reactive oxygen species and antioxidant machinery in abiotic stress tolerance in crop plants. Plant Physiol. Biochem..

[63] Shah T., Latif S., Saeed F., Ali I., Ullah S., Alsahli A.A., Jan S., Ahmad P. (2021). Seed priming with titanium dioxide nanoparticles enhances seed vigor, leaf water status, and antioxidant enzyme activities in maize (*Zea mays* L.) under salinity stress. J. King Saud Univ. -Sci..

[64] Mozafari A.A., Ghadakchi asl A., Ghaderi N. (2018). Grape response to salinity stress and role of iron nanoparticle and potassium silicate to mitigate salt induced damage under in vitro conditions. Physiol. Mol. Biol. Plants.

[65] Dionisio-Sese M.L., Tobita S. (1998). Antioxidant responses of rice seedlings to salinity stress. Plant Sci..

[66] Foyer C., Lelandais M., Galap C., Kunert K.J. (1991). Effects of elevated cytosolic glutathione reductase activity on the cellular glutathione pool and photosynthesis in leaves under normal and stress conditions. Plant Physiol..

[67] Omar S.A., Elsheery N.I., Pashkovskiy P., Kuznetsov V., Allakhverdiev S.I., Zedan A.M. (2023). Impact of titanium oxide nanoparticles on growth, pigment content, membrane stability, DNA damage, and stress-related gene expression in Vicia faba under saline conditions. Horticulturae.

[68] Tichá T., Samakovli D., Kuchařová A., Vavrdová T., Šamaj J. (2020). Multifaceted roles of HEAT SHOCK PROTEIN 90 molecular chaperones in plant development. J. Exp. Bot..

[69] Cox A., Venkatachalam P., Sahi S., Sharma N. (2016). Silver and titanium dioxide nanoparticle toxicity in plants: A review of current research. Plant Physiol. Biochem..

[70] Demir E., Kaya N., Kaya B. (2014). Genotoxic effects of zinc oxide and titanium dioxide nanoparticles on root meristem cells of Allium cepa by comet assay. Turk. J. Biol..

