# Supplementary material for: Employing Titanium Dioxide Nanoparticles as Biostimulant against Salinity: Improving Antioxidative Defense and Reactive Oxygen Species Balancing in Eggplant Seedlings
Source: Antioxidants (Basel). 2024 Oct 8;13(10):1209. doi: 10.3390/antiox13101209 (PMC11505378; doi:10.3390/antiox13101209)
Supplement: Supplementary file 1 [file antioxidants-13-01209-s001.zip › antioxidants-3213839-supplementary.pdf]

Table S1. Analysis of Variance for eggplants seedlings under salinity stress with foliar application of TiO_2-NPs.

Ascorbate peroxidase			
**Source of variance**	**Degree of Freedom**	**SS**	**MS**
**Treatment**	2	459.26	229.628**
**Stress**	2	1371.08	685.542**
**Treatment*Stress**	4	121.18	30.296*
**Error**	18	169.94	9.441
**Total**	26	2121.47	
**Catalase**			
**Source of variance**	**Degree of Freedom**	**SS**	**MS**
**Treatment**	2	345.35	172.67**
**Stress**	2	2630.51	1315.26**
**Treatment*Stress**	4	49.11	12.28
**Error**	18	260.73	14.48
**Total**	26	3285.70	
**Dry Biomass**			
**Source of variance**	**Degree of Freedom**	**SS**	**MS**
**Treatment**	2	169.280	84.64**
**Stress**	2	164.287	82.1433**
**Treatment*Stress**	4	5.953	1.4883
**Error**	18	166.207	9.2337
**Total**	26	505.727	
**Fresh Biomass**			
**Source of variance**	**Degree of Freedom**	**SS**	**MS**
**Treatment**	2	1594.60	797.3**
**Stress**	2	3937.07	1968.53**
**Treatment*Stress**	4	54.83	13.71
**Error**	18	364.79	20.27
**Total**	26	5951.28	
**Glutathione reductase**			
**Source of variance**	**Degree of Freedom**	**SS**	**MS**

**Treatment**	2	36.964	18.482**
**Stress**	2	227.811	113.906**
**Treatment*Stress**	4	43.804	10.951**
**Error**	18	11.628	0.646
**Total**	26	320.208	
**Hydrogen peroxide**			
**Source of variance**	**Degree of Freedom**	**SS**	**MS**
**Treatment**	2	491.58	245.79**
**Stress**	2	2850.66	1425.33*
**Treatment*Stress**	4	212.69	53.17**
**Error**	18	116.40	6.47
**Total**	26	3671.34	
**Malondialdehyde**			
**Source of variance**	**Degree of Freedom**	**SS**	**MS**
**Treatment**	2	78.088	39.044**
**Stress**	2	361.702	180.851**
**Treatment*Stress**	4	21.201	5.3*
**Error**	18	28.525	1.585
**Total**	26	489.515	
**Number of leaves**			
**Source of variance**	**Degree of Freedom**	**SS**	**MS**
**Treatment**	2	36.741	18.3704
**Stress**	2	168.074	84..037**
**Treatment*Stress**	4	9.926	2.4815
**Error**	18	102.667	5.7037
**Total**	26	317.407	
**Non-photochemical quenching**			
**Source of variance**	**Degree of Freedom**	**SS**	**MS**
**Treatment**	2	1.7947	0.897**
**Stress**	2	7.8451	3.922**
**Treatment*Stress**	4	0.4727	0.118**
**Error**	18	0.2289	0.012
**Total**	26	10.3414	

Sodium ion			
**Source of variance**	**Degree of Freedom**	**SS**	**MS**
**Treatment**	2	159.76	79.88**
**Stress**	2	3329.05	1664.52**
**Treatment*Stress**	4	112.44	28.11
**Error**	18	119.66	6.65
**Total**	26	3720.91	
**Peroxidase**			
**Source of variance**	**Degree of Freedom**	**SS**	**MS**
**Treatment**	2	3681.56	1840.78**
**Stress**	2	3449.56	1724.78**
**Treatment*Stress**	4	473.56	118.39
**Error**	18	471.33	26.19
**Total**	26	8076.00	
**Quantum yield (Day)**			
**Source of variance**	**Degree of Freedom**	**SS**	**MS**
**Treatment**	2	0.01709	0.00854**
**Stress**	2	0.23807	0.11903**
**Treatment*Stress**	4	0.02111	0.00528**
**Error**	18	0.00200	0.00011
**Total**	26	0.27827	
**Quantum yield (Night)**			
**Source of variance**	**Degree of Freedom**	**SS**	**MS**
**Treatment**	2	0.03850	0.01925**
**Stress**	2	0.59521	0.29760**
**Treatment*Stress**	4	0.03181	0.00795**
**Error**	18	0.00207	0.00011
**Total**	26	0.66759	
**Root length**			
**Source of variance**	**Degree of Freedom**	**SS**	**MS**
**Treatment**	2	105.41	52.704
**Stress**	2	858.96	429.481**
**Treatment*Stress**	4	31.48	7.870

**Error**	18	405.33	22.519
**Total**	26	1401.19	
**Shoot length**			
**Source of variance**	**Degree of Freedom**	**SS**	**MS**
**Treatment**	2	9.185	4.5926
**Stress**	2	68.963	34.4815**
**Treatment*Stress**	4	2.370	0.5926
**Error**	18	96.000	5.3333
**Total**	26	176.519	
**Superoxide dismutase**			
**Source of variance**	**Degree of Freedom**	**SS**	**MS**
**Treatment**	2	920.89	460.44**
**Stress**	2	8094.89	4047.44**
**Treatment*Stress**	4	68.89	17.22
**Error**	18	522.00	29.00
**Total**	26	9606.67	
**Leaf greenness (SPAD)**			
**Source of variance**	**Degree of Freedom**	**SS**	**MS**
**Treatment**	2	422.30	211.15**
**Stress**	2	2275.85	1137.93**
**Treatment*Stress**	4	273.70	68.43**
**Error**	18	142.67	7.93
**Total**	26	3114.52	
**Total soluble proteins**			
**Source of variance**	**Degree of Freedom**	**SS**	**MS**
**Treatment**	2	0.3863	0.19317
**Stress**	2	16.2387	8.11935**
**Treatment*Stress**	4	0.3168	0.07921
**Error**	18	2.0136	0.11187
**Total**	26	18.9555	

* $p < 0.05$ and ** $p < 0.01$.

Table S2. Principal Components for physiological and biochemical responses in the leaves of eggplant seedlings under salinity with application of TiO_2-NPs seedlings under salinity.

Variables	PC1	PC2
**RL**	-0.24	0.09
**SL**	-0.25	0.06
**NOL**	-0.25	0.15
**FB**	-0.24	0.2
**DB**	-0.21	0.24
**SPAD**	-0.24	0.16
**QY-D**	-0.25	0.04
**QY-L**	-0.25	0.07
**NPQ**	0.25	-0.17
**SOD**	0.22	0.29
**POD**	0.14	0.49
**CAT**	0.22	0.3
**H2O2**	0.24	-0.15
**TSP**	0.25	0.11
**MDA**	0.25	-0.17
**APX**	0.19	0.41
**GR**	0.2	0.36
**Na+**	0.25	-0.04
**Standard deviation**	3.77	1.65
**Variability (%)**	58.3%	13.9%
**Cumulative (%)**	58.3%	72.2%

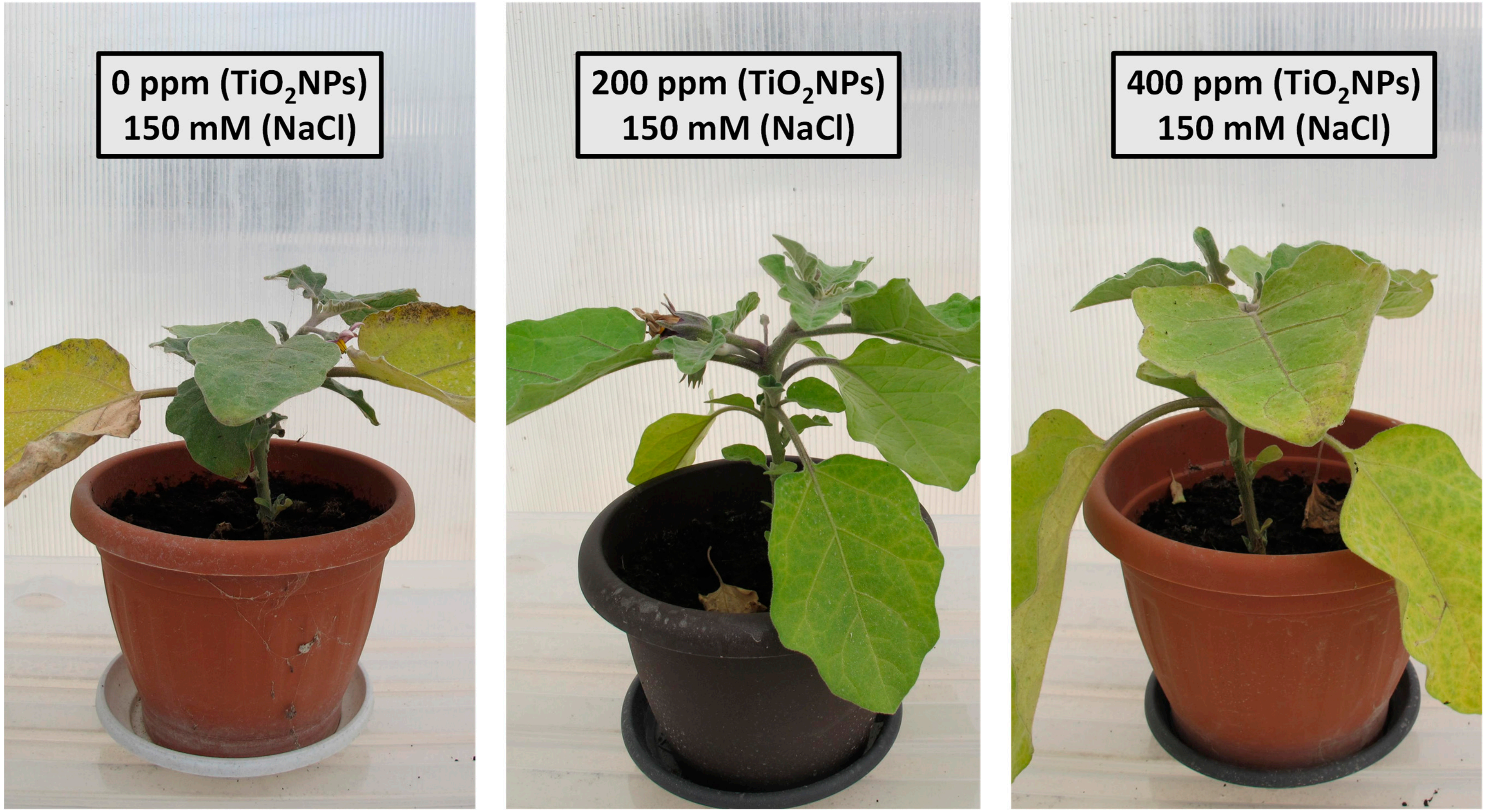

Figure S1. Effect of TiO_2NPs on eggplant seedlings at 150 mM NaCl.
